# Economic globalization, nutrition and health: a review of quantitative evidence

**DOI:** 10.1186/s12992-019-0456-z

**Published:** 2019-02-20

**Authors:** Soledad Cuevas García-Dorado, Laura Cornselsen, Richard Smith, Helen Walls

**Affiliations:** 1 Department of Global Health and Development, London School of Hygiene and Tropical Medicine, Tavistock Place, London, WC1H 9SH UK; 2 Leverhulme Centre for Integrate Research on Agriculture and Health, 36 Gordon Square, London, WC1H 0PD UK; 3 University of Exeter, Stocker Rd, Exeter, EX4 4PY UK; 4 SOAS, University of London, Bloomsbury, London, WC1H 0XG UK

**Keywords:** Globalization, Trade liberalization, FDI, Nutrition transition, Non-communicable disease

## Abstract

**Background:**

Unhealthy dietary patterns have in recent decades contributed to an endemic-level burden from non-communicable disease (NCDs) in high-income countries. In low- and middle-income countries rapid changes in diets are also increasingly linked to malnutrition in all its forms as persistent undernutrition and micronutrient deficiencies continue to coexist with a rising prevalence of obesity and associated NCDs. Economic globalization and trade liberalization have been identified as potentially important factors driving these trends, but the mechanisms, pathways and actual impact are subject to continued debate.

**Methods:**

We use a ‘rigorous review’ to synthesize evidence from empirical quantitative studies analysing the links between economic globalization processes and nutritional outcomes, with a focus on impact as well as improving the understanding of the main underlying mechanisms and their interactions.

**Findings:**

While the literature remains mixed regarding the impacts of overall globalization, trade liberalization or economic globalization on nutritional outcomes, it is possible to identify different patterns of association and impact across specific sub-components of globalization processes. Although results depend on the context and methods of analysis, foreign direct investment (FDI) appears to be more clearly associated with increases in overnutrition and NCD prevalence than to changes in undernutrition. Existing evidence does not clearly show associations between trade liberalization and NCD prevalence, but there is some evidence of a broad association with improved dietary quality and reductions in undernutrition. Socio-cultural aspects of globalization appear to play an important yet under-studied role, with potential associations with increased prevalence of overweight and obesity. The limited evidence available also suggests that the association between trade liberalization or globalization and nutritional outcomes might differ substantially across population sub-groups.

Overall, our findings suggest that policymakers do not necessarily face a trade-off when considering the implications of trade or economic liberalization for malnutrition in all its forms. On the contrary, a combination of nutrition-sensitive trade policy and adequate regulation of FDI could help reduce all forms of malnutrition. In the context of trade negotiations and agreements it is fundamental, therefore, to protect the policy space for governments to adopt nutrition-sensitive interventions.

**Electronic supplementary material:**

The online version of this article (10.1186/s12992-019-0456-z) contains supplementary material, which is available to authorized users.

## Introduction

International trade as a proportion of global GDP has almost doubled since the beginning of the 1970s, and now represents almost 60% of world GDP [1]. This increased exchange of goods and services has occurred as part of a wider process of globalization, encompassing inter-related economic, social and cultural components [2]. Trade policies and globalization processes are deeply transforming societies, shaping political institutions, economic and social relationships, modes of production, consumption patterns and lifestyles. These structural factors are increasingly recognized as important drivers of nutrition and health outcomes [3–5]. In particular, trade reforms and liberalization have often been linked to both under-nutrition and the rapid rise in overweight and obesity and spread of diet-related non-communicable diseases (NCDs) in low- and middle-income countries (LMICs) [6, 7]. Traditionally considered a problem of high-income countries, the burden of overweight, obesity and diet-related NCDs has in recent years greatly increased in LMICs, which already account for more than 80% of deaths from NCDs worldwide [8]. Increased prevalence of overweight, obesity and NCDs, however, often coexists with persistent undernutrition and micronutrient deficiency, leading to what is known as a double (or triple) burden of malnutrition [9].

Debate on the links between trade liberalization and nutrition can be traced back to the controversial implementation of structural adjustment programmes by the World Bank and International Monetary Fund (IMF) in the 1980s [10, 11] . Following the international food crisis in 2008 and in the context of the growing obesity “epidemic”, however, this issue has gained renewed attention from researchers and policy-makers. This has led to the recent surge of publications that approach the issue, and increasingly so from different angles, providing new and updated evidence on the subject.

Several recent reviews have mapped the pathways between trade agreements and food-related aspects of public health, including related to food environments [12], and the nutrition transition [13]. Studies have synthesized existing evidence of the impacts of agricultural trade liberalization on food security in LMICs [14], and analysed the effect of trade and investment liberalization on prevalence of NCDs in Asia [15]. There is a wide variation in terms of quality and design of the studies included in these reviews, ranging from case-studies to quantitative multi-country and natural experimental designs. In addition, Barlow et al. [16] recently published a more general review of quantitative studies analysing the impact of regional trade agreements on major health risk factors and outcomes, including some evidence on nutrition-related outcomes.

To our knowledge, however, there has not been a systematic analysis and synthesis of the empirical evidence on the associations between economic globalization and liberalization processes and nutrition outcomes. This review complements the existing evidence, through the use of a ‘rigorous review’ methodology as described by Hagen-Zanker and Mallett [17] to undertake analysis of studies quantifying the relationship between economic globalization and nutritional outcomes including under and overnutrition and incorporating new, relevant evidence not covered by previous reviews. The specific focus on malnutrition in all its forms is in line with recent literature calling for integrated approaches to address the growing double (or triple) burden of malnutrition [18, 19]. Malnutrition in all its forms is understood to include undernutrition, micronutrient deficiencies, overweight and obesity and related NCDs [20]. This approach allows us also to explore evidence of the overlapping processes of dietary convergence-divergence that take place as food systems become increasingly integrated.

## Conceptual framework

Jenkins (2004) describes globalization as “a process of greater integration within the world economy, through movements of goods and services, capital, technology and (to a lesser extent) labour, which leads increasingly to economic decisions being influenced by global conditions” [21]. This definition focuses on economic globalization, concerned with changes taking place to world trade and investment, but adopting the view that economic forces underlie and shape the overall globalization process, connecting what are sometimes described as different aspects of globalization, including socio-cultural changes and information flows [2].

**Fig. 1:**
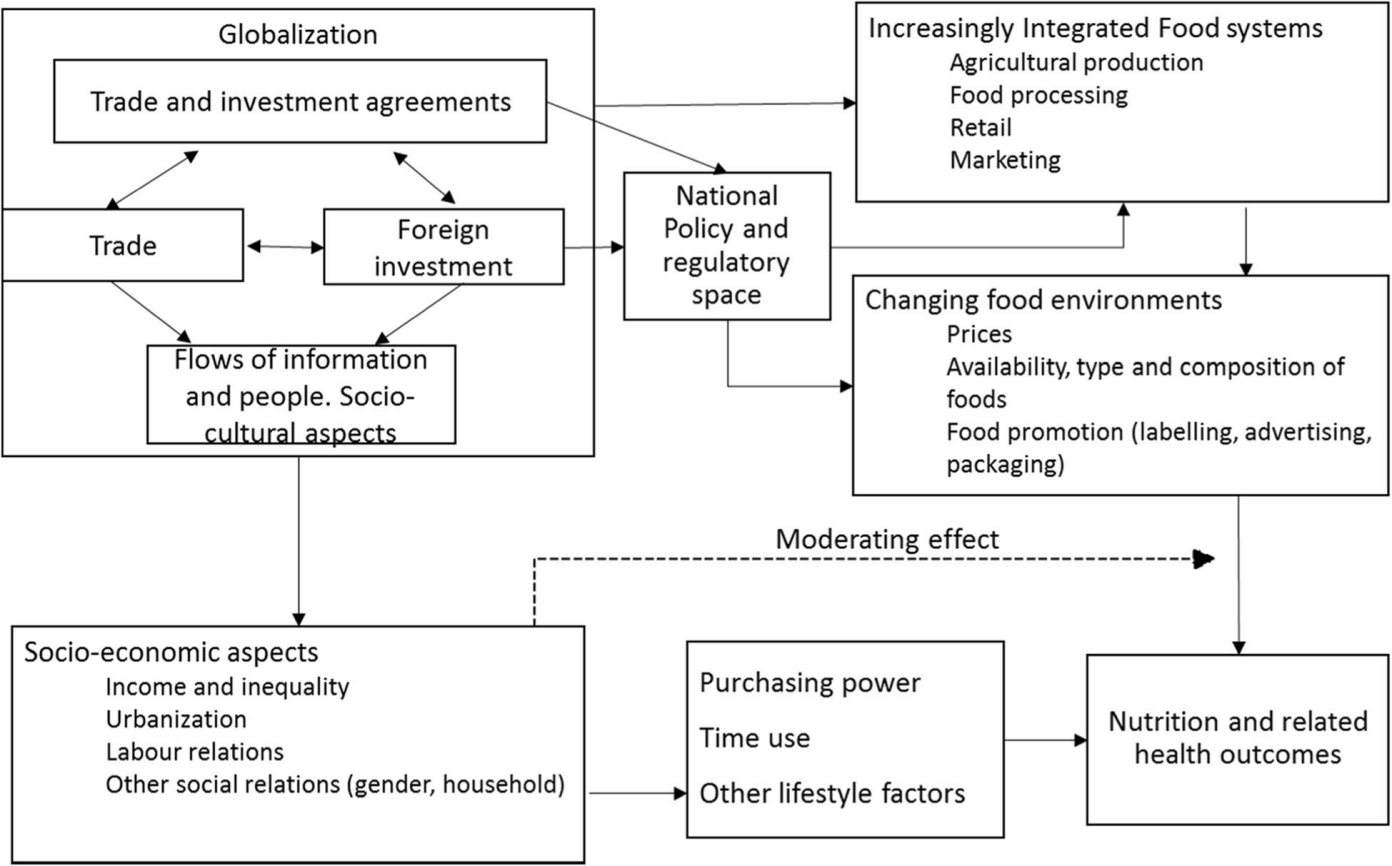
Conceptual framework of the relationship between globalization, nutrition and related health outcomes. Synthesised based on the frameworks of [2, 12, 14]

We have developed a framework, shown in Fig. 1, to conceptualise the relationships between globalization, nutrition and related health outcomes. The framework, informed by existing theoretical works and published conceptual frameworks, ([2, 4, 6, 12, 22]) includes the main sub-components of globalization and the trade and investment policies underpinning the process. It depicts the impact of globalization processes on nutrition outcomes as linked through changes in food systems and food environments, as well as through impacts on national policy and regulatory space, and through the transformation of broader socio-economic factors. Socio-economic factors also play an important role as mediators of the effect of food environment changes, resulting in heterogeneous effects across population sub-groups. Before proceeding to a description of the method used and our study findings, we will briefly describe each of the domains in Fig. 1, as they relate to the wider framework.

### International trade and food environments

This pathway is shown at the top and to the right in our conceptual framework. International trade is generally understood to encompass the exchange of both goods and services across countries. Although most of the papers included in this review tend to focus their discussion on trade in goods rather than services, perhaps implicitly assuming more relevant linkages between trade in goods and dietary and nutrition outcomes, many use composite indices that include trade in services, such as the economic component of the KOF index for globalization or its sub-components.^1^

The creation of a global market for food products has important effects on the availability and prices of food commodities. On the production side, global markets encourage specialization in export crops, which tends to create economies of scale in agricultural and food production, leading to increased global output, but also to homogenization in the availability of food products [7, 23, 24]. On the demand side, countries can increase their access to a variety of goods through imports, including essential foodstuffs [25] and healthy foods [26] as well as potentially unhealthy processed and ultra-processed products [27, 28]. The relationship between international trade and food prices is complex. Access to international commodity markets can reduce food price volatility by diminishing the effect of local shocks. However, it increases the exposure to global demand instability, as well as to volatility in the “terms of trade” for highly specialized countries [29]. On average, trade openness has been found to lower the relative price of calorie-dense foods and animal feed [30].

### Foreign direct investment

Foreign direct investment (FDI) is an investment by which a foreign company acquires control over a (new or pre-existing) business. This is to distinguish FDI from portfolio investments where investors are not involved in or have control over the day to day operations of a business [31] Like trade, FDI is also thought to play an important role in transforming food systems. It is FDI, rather than trade, that is considered to be the currently preferred method for Transnational Food Companies (TFC) to enter new markets for processed foods, allowing multinationals to advertise and market their products more efficiently, creating a demand while, simultaneously, adapting to consumer characteristics [32].

Both FDI and advertising are also thought to lead to indirect effects on nutrition; increasing competition among local firms and increasing the demand not only for the marketed brand, but for the whole category, be it snacks, ice-cream or “diet” and “wellness” products [6]. Additionally, retail and marketing strategies contribute to market segmentation, which is believed to lead to a divergence in dietary patterns within countries, even as diets converge across countries. [6, 33, 34].

### Sociocultural aspects of globalization

Increased global flows of information (and people) can transform cultural norms, social relations, and consumption patterns. The spread of communication technology and infrastructure makes it possible for information to be shared more widely and faster, but it does not in itself explain the content, influence and directionality of the information exchange. These are thought to be driven by economic forces operating through the expansion of large multinationals in media, communications and marketing [35]. The globalization of marketing and promotion, aided by the expansion of TFC and global marketing companies, are thought to play an important role in the integration of food markets, changing consumption patterns, and creation of a demand for new products and brands [36].

### Policy and regulatory space

The creation of progressively integrated global markets is underpinned by trade and investment agreements and policies. The World Trade Organization (WTO) remains the main international organization responsible for the global rules of trade between countries.^2^ Since the early 1990s however, an increasing number of regional and bilateral trade agreements have been negotiated outside of the WTO system.^3^ These agreements frequently reflect power imbalances between participating countries, can be heavily influenced by the interests of multinational companies and can have deep impacts on domestic policy [37, 38]. The inclusion of mechanisms for investor-state dispute settlement, whereby companies can directly sue states, is an example of the new ways in which this “new generation” of agreements can reduce the capacity of governments to implement health-oriented regulation that might lead to reduced profits for foreign investors [15, 39, 40]. Some authors have specifically argued that trade and investment agreements can negatively affect nutritional outcomes by directly reducing the regulatory and policy space for health-promoting initiatives [40, 41] . We have found a small number of studies that quantitatively analysed aspects of political globalization alongside measures of economic dimensions. However, these are very partial and non-specific measures of the potential impacts of trade agreements on the policy space. It is important to bear in mind that some of the most influential literature on this topic [39, 41] is qualitative and was not included in this review as our focus is specifically on quantitative studies. This literature, however, does suggest that the impact of restrictions to the policy space, associated with trade liberalization processes, should not be underestimated, as it can curtail the capacity of governments to protect public health [42].

### Interaction with socioeconomic drivers of nutrition

Market integration and trade and investment agreements not only affect nutrition outcomes through their impacts on the food sector. Globalization processes deeply transform all aspects of society, in ways which can indirectly affect nutrition outcomes. Globalization has been found to be associated with GDP and income growth [43, 44], but also to increased income inequality [45], as well as to [46] urbanization [47, 48]. According to some authors, globalization has also been associated with a deterioration in labour standards and conditions [49], coupled with a transition towards sedentary and “knowledge-based” work [50] while, for others, integration in the global economy increases the returns to labour, encouraging larger investments in health [51]. Although some mechanisms are better understood than others, all of these structural socioeconomic changes have been linked to changes in dietary patterns and should be taken into account when assessing the links between globalization and nutrition outcomes.

## Methods

### Methodological approach

Systematic review methods have recently been subject to criticism regarding their inflexible application to social sciences. Critics have pointed out the considerable degree of subjectivity in the interpretation, definition and use of concepts in social sciences, as well as the importance of context, which is often ignored in traditional systematic reviews [17, 52]. Similar arguments have been made specifically concerning reviews in public health [53, 54]. Considering this, we undertook a ‘rigorous review’, following the core principles listed in Hagen-Zanker and Mallet [17] as guidance on conducting rigorous, evidence-focused literature reviews in international development. Thus, we adhered to the principles of rigour, transparency and replicability at the core of the systematic literature review process, but followed a process that also allows for flexibility and reflexivity [17] . Importantly, in our analysis we acknowledge the subjectivity in interpretation of concepts and thus emphasise the importance of context in the interpretation of the studies and their significance for policy-making. Furthermore, our focus is on “how” social change works, rather than on “what” the impact of any policy or process is.

The rigorous review approach has also allowed us to classify the included articles according to relevant criteria (see Table 2), facilitating a structured analysis and discussion of the findings in the literature.

### Search

We searched for studies containing terms related to economic globalization, trade and investment liberalization, food and food environments, and nutrition and related health outcomes as well as terms related to quantitative research methods. We conducted this search in five databases (Web of Science, Scopus, Global Health, EconLit and MEDLINE) and several institutional websites, including WHO, WTO, UNCTAD, IFPRI and USAID. We complemented this with a general search on Google and Google Scholar. Searches were carried out in March-2017. We checked the reference lists of articles selected for full text review for further relevant publications.

**Fig. 2:**
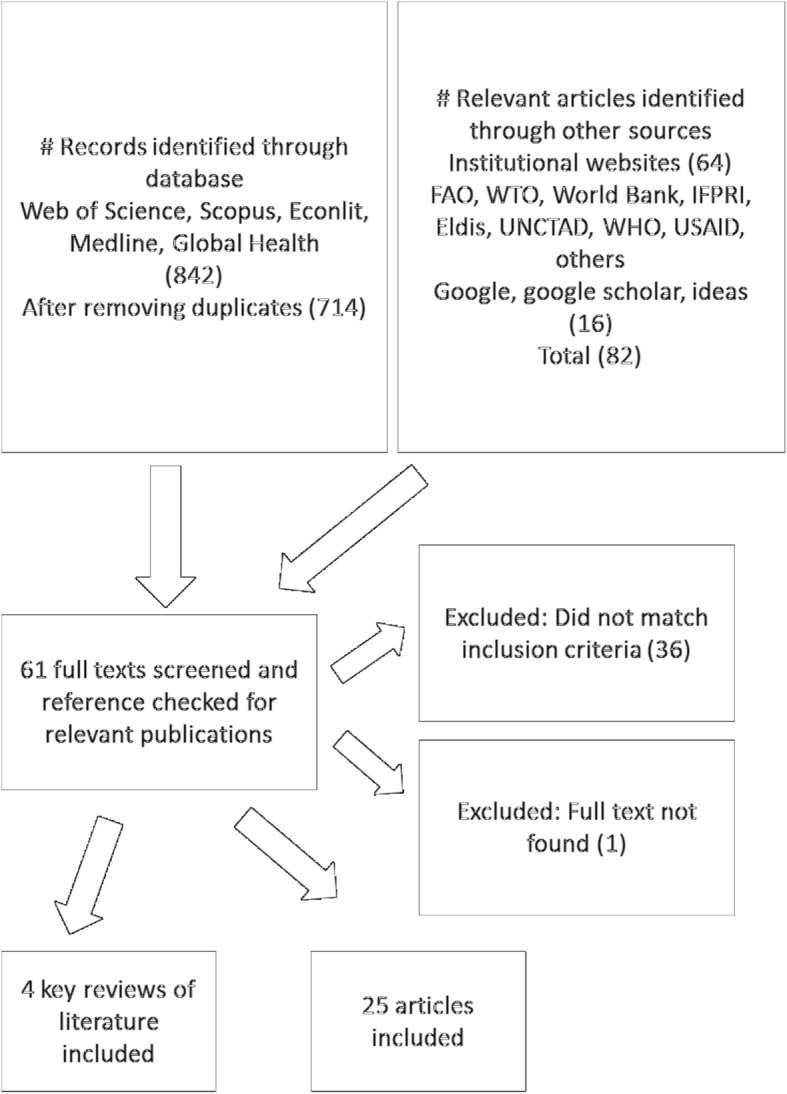
Document flow diagram

The references were screened by two authors and any disagreements were resolved through discussion. In the first round of screening, potentially relevant articles were selected based on the general focus of the study as judged by the title and abstract. In the second round, relevant references were screened based on inclusion criteria, described in Table 1. Figure 2 shows the document flow and the number of references retrieved in the different stages of the search and screening process [6]. An additional Contains assessment criteria provides further detail of the search strategy [see Additional file 1].

**Table 1:** Inclusion criteria

Focus Includes: Studies that retrospectively analyse the impacts of economic globalization processes on nutrition and related health outcomes, both in high, medium and low income countries.	
Methods Includes: Quantitative, empirical studies that analyse associations between economic globalization and nutrition and related health outcomes (e.g. multi-country regression analysis controlling for covariates or country heterogeneity, multi-level regression, quasi-experimental designs, time series analysis). Excludes: Prospective simulation based analysis, qualitative studies, studies that use quantitative information descriptively, without statistical analysis.	
Outcomes Includes: Diet-related health outcomes (e.g. diabetes, CVD). Measures and proxies for nutrition outcomes (e.g. anthropometric measurements, body mass index, food and nutrient intake, availability or supply of foods or nutrients in context specific cases (e.g. availability/supply of any foods/nutrients in undernutrition context or availability/supply of unhealthy foods (clearly defined) in any context). Excludes: Health outcomes that cannot be linked to nutrition; mortality and life expectancy outcomes (cannot be linked directly to nutrition); supply of food (nutrients) without clear link to nutrition in the population context.	
Definitions Includes: Studies looking at trade flows, tariff changes, trade and investment agreements or policies, trade openness; measures of economic globalization. We do not include studies that focus exclusively on global flows of information, social or cultural globalization. Excludes: Studies analysing the impacts of policies or agreements that might be affected by trade negotiations (e.g. national agricultural or monetary policy); impact of measures introduced to counteract the effects of trade liberalization, such as export bans.	
Year and language of publication Includes: articles published from January 1990 in English language.

### Inclusion criteria

Detailed explanation of inclusion criteria is provided in Table 1. The criteria take into account the overall focus of the paper, methods, definition of globalization and nutrition outcomes, and the year and language of the publication.

### Information extraction and analysis

Articles meeting the inclusion criteria were recorded in an Excel database including key information on context (country, time frame), globalization processes observed (including definitions of the processes), type and source of data analysed, statistical methods applied, and main findings and conclusions from the study. The analysis of the studies included examining findings against existing conceptual frameworks and theoretical evidence, as well as with the findings of previous reviews on similar topics.

## Results

Seven hundred fourteen articles were identified from five different databases, another 64 were retrieved from institutional websites, and 16 from additional searches on Google or Google scholar. The abstracts of all studies were screened and the full texts of 63 studies which were found to be relevant were downloaded for screening. 24 of these met our inclusion criteria. In addition, four relevant review studies were identified.

Of the 24 articles included, 11 look at diet-related health outcomes or biomarkers, including underweight, overweight, obesity, diabetes, CVD prevalence and BMI. A further 13 articles used context-relevant proxies of nutrition outcomes, including energy (kcal) intake per day, dietary diversity, and markers of dietary quality such as consumption of unhealthy food commodities, fat intake, consumption of protein and animal protein. Half of the studies (12 out of 24) focussed on LMICs. Most studies used country level data, while only three studies used multi-level models to account for effects occurring at different levels of aggregation. Natural experiments or difference-in-difference designs were used in three studies, and one study relied on single-country time series data. Two studies used less conventional approaches such as non-parametric correlation or structural equation modelling. Details of variables used, study design, data sources and main findings are provided in Table 2.

**Table 2:** Included articles

	Included Articles	Methods	Definition of trade liberalization	Outcome variable	Region	Years	Main findings	Type of evidence
1	(de Soysa and de Soysa, 2017)	Multivariate regression using country-level panel data	KOF index of globalization. Analyse trade openness and FDI components separately	Prevalence of obesity in young people aged 2–19 from GBD study	180 countries	1990–2013	Trade openness and economic globalization result in lower obesity rates among the younger groups of population	BABCA
2	(Oberländer, Disdier, and Etilé, (2016)	Multivariate regression using country-level panel data	KOF index of globalization. Economic and social components analysed separately	Prevalence of diabetes; BMI; markers of dietary quality (animal protein, free fat, sugar)	70 countries	1970–2011	Economic globalization negatively impacts health outcomes; socio-cultural globalization increases supplies of animal protein and sugar	BAACA
3	(Costa-Font and Mas, (2016)	Multivariate regression using country-level panel data	KOF index of globaliztion; economic and social components analysed separately; CSGR index	Prevalence of obesity	26 HIC	1989–2004	Globalization significantly increases obesity; economic component reduces obesity (effect non-significant when accounting for various controls and potential mechanisms); social component increases obesity	BAACA
4	Goryakin et al., (2015)	Multi-country multi-level panel data controlling for both individual and country-level covariates.	KOF index of globalization and sub-components (economic, political, social)	Overweight and obesity	56 countries	1991–2009	Globalization increases overweight, but the social and political components are the most relevant	ABBAA
5	Miljkovic et al., (2015)	Multivariate regression using country-level panel data	FDI; trade openness; Global Socialization Index (GSI)	Prevalence of obesity	76 countries	1986–2008	Trade openness increases obesity in the fixed effects specification, but not in the quantile regression. FDI and GSI increase obesity for least developed countries, where obesity rates are low	BABCB
6	Sudharsanan,et al., (2015)	Non-parametric correlation and multivariate first-difference regression estimates	FDI	prevalence of diabetes in 10-year age groups	both HIC and LMIC	1990, 2000, 2008	Once ageing in population is taken into account, there is no evidence of FDI or other macroeconomic variables such as GDP, having an influence on prevalence of diabetes	BABAC
7	Nandi et al., (2014)	Meta-regression using multi-country cross-sectional individual level data.	Mean tariff percentage averaged 1990–1999. FDI	BMI; odds of being underweight, overweight and obese at the individual level for women in LMIC	40 LMIC	2002–2003	Tariff reduction is associated with lower odds of being underweight. FDI is associated with higher odds of being overweight among rural men only. Higher income is associated to higher odds of being overweight	CAAAC
8	Neuman et al. (2014)	Multi-level modelling using cross-sectional data	FDI, mean tariff levels	BMI; over and under-weight	38 LMIC	1991, 2010	FDI is positively associated with BMI among poorest respondents in rural areas	BAAAB
9	Vogli, R. de et al., (2014)	Multivariate regression using country-level panel data.	KOF index of globalization (economic component)	BMI	127 countries	1980–2008	Globalization is positively associated to an increased BMI. Inequality also shows a positive association in high-income countries	BABCA
10	Schram, Labonte, and Sanders (2013)	Trend analysis and Structural Equation Modelling using multi-country cross-sectional data	KOF index of economic globalization	CVD, overweight, obesity	39 countries	2008 for SEM	Economic globalization negatively impacts all health outcomes.	CAACC
Context-relevant proxies of nutrition outcomes								
11	Jenkins and Scanlan (2001)	Multivariate regression analysis with country-level panel data.	Foreign investment, dependence on primary exports	Child undernutrition (weight-for-age), per capita energy and protein availability	88 less developed countries	1970–1990	There is a negative association between dependence on non-service exports and energy supply but this is non-significant after controlling for other economic variables. Neither FDI nor export dependence have an impact on child underweight	BAACA
12	Dithmer and Abdulai (2017)	Multivariate regression using country-level panel data.	Trade openness	energy consumption; diet diversity; diet quality	151 countries	1980–2007	Trade openness increases average dietary energy consumption, dietary diversity and indicators of dietary quality	BAACA
13	Baker et al. (2016)	Difference-in-difference/Natural experiment	Ratification and enforcement of FTA with US	per capita sales of soft drinks	Peru	1999–2013	The study finds a diversification of soft drinks. Sales of carbonated drinks stagnate, but bottled water, sports and energy drinks increase	AACCC
14	Schram A, Labonte R et al., (2015)	Difference-in-difference/Natural experiment	Adoption of trade agreement, FDI	Consumption of carbonated beverages	Vietnam and Philippines	1995–2012	The adoption of a trade agreement increases per-capita sales of beverages	AACCA
15	Ogundari, (2015)	Multivariate regression using country-level panel data.	Trade openness	Nutrient supply, calories, proteins, fat	43 countries	1975–2009	Trade openness seems to contribute to nutrient supply convergence in Sub-Saharan Africa	BACCA
16	Zakaria (2014)	Multivariate regression analysis using country-level panel data	Trade openness	Per capita availability of energy, fat	5 South Asian countries	1972–2013	Trade openness and tariff reductions are associated with increased energy availability per capita	BACCA
17	Bezuneh and Yiheyis, (2014)	Multivariate regression analysis using country-level panel data	Implementation of liberalization policies (defined through dummy variables)	Per capita dietary energy supply	37 developing countries	1980–2000	The removal of trade barriers is associated to short-term falls in nutrient availability per capita, with positive longer-term effects and insignificant “net” impacts	BACCC
18	Stuckler et al. (2012)	Multivariate regression analysis.	FDI, trade agreement with US	Sales per capita of sugar-sweetened beverages (SSB)	44 LMIC	1997–2010?	Both FDI and trade agreements with US increase sales per capita of SSB. Economic growth in the absence of FDI does not increase sales of SSB	BACCC
19	Djokoto (2012)	Cointegration analysis, time series using country-level data	FDI into agricultural sector	Per capita dietary energy supply	Ghana		FDI into the agricultural sector is detrimental for food security in Ghana	BACCC
20	Mihalache and O’Keefe (2011)	Cointegration analysis, time series using country-level data	FDI into primary sector, manufacturing and service sector	Per capita dietary energy supply	56 LMIC	1981–2001	FDI into the primary sector is detrimental for food security. FDI into manufacturing improves food security, FDI into services has ambiguous effects	BACCA
21	Del Ninno and Dorosh (2003)	Natural experiment. The authors compare three episodes of intense floods, their impact on crops, availability and price of rice, and calorie intake of affected households compared to those not affected	Liberalization of private-sector rice imports from India, in the early 1990s	Daily energy intake per capita	Bangladesh	1977, 1988, 1998	In the absence of private sector imports, per capita consumption of the rural poor would have decreased by 44 to 109 Kcal/Day, (out of an average of 1636). Public interventions including price stabilization and transfers also play an important role	AACCA
22	Wimberley and Bello (1992)	Multivariate regression analysis using country-level panel data	Primary export dependence; Transnational Company (TNC) investment	Per capita energy, and protein availability, total and from vegetable sources	59 third world countries	1967–1985	There is evidence of a negative association between FDI and nutrition-related outcomes in developing countries, as well as a much smaller negative association for dependence on non-service exports	BACCA
23	Wimberley (1991)	Multivariate regression analysis using country-level panel data	TNC investment	Per capita energy and protein availability	60 Third World Countries	1970–1985	There is a strong negative association between FDI and per capita availability of energy and protein in developing countries	BACCA
24	Gacitúa & Bello (1991)	Multivariate regression analysis using country-level panel data	Non-service exports as a proportion of GDP	Per capita energy, protein availability total and from vegetable; Z-score standardized measure of calorie and protein consumption	15 Latin-American Countries	1967–1985	This study finds a negative association between dependence on non-service exports and per capita supply of energy and proteins in Latin America	BACCC
Key literature reviews								
1	Barlow et al., (2017)	Systematic review	Adoption of trade and investment agreements	Health outcomes, risk factors		–	Trade and investment agreements can increase risk factors for NCD (beverage consumption) while also affecting protective factors (public health policies). However, certain agreements can increase access to patented medicines, with positive impacts on health	
2	Baker P, Kay A, Walls H. (2014)	Semi-structured review	Trade liberalization, trade and investment agreements, others	prevalence of NCDs and main risk factors	ASEAN+ 3, India	–	Trade liberalization can promote NCD through two main pathways: increasing access to unhealthy products and constraining governments’ space to promote health	
3	Friel et al., (2013)	Review of literature and pathway mapping	Trade liberalization, trade and investment agreements, others	NCDs, obesity	Not restricted	–	The authors identify several pathways through which trade liberalization can affect NCD	
4	McCorriston S et al. (2013)	Systematic Review	Various. Trade and related policies	Food Security	Developing Countries	–	The authors find mixed evidence and a strong context-dependence of associations and impacts	

Type of evidence. Design (A: natural experiment, B:longitudinal/time-series-cross-sectional (TSCS) or time series, C: cross-sectional); Statistical analysis (A: structural equation modelling, reduced-form regression, time-series analysis, other, B: simple one-on-one correlations, C: descriptive) Type of outcome variable (A: Uses several related outcome variables including prevalence or status of dietary-related disease as well as relevant proxies, B: Nutrition outcomes: Prevalence/status of diet-related disease (CVD, diabetes or others). Relevant biomarkers (obesity, underweight or overweight, BMI), C: Context-relevant proxies for nutrition outcomes: per capita consumption of key foods/nutrients); Data (A: Both individual and country-level outcome variables, B: Individual-level outcomes, C: Country-level outcome variables); Sensitivity analysis (A: Thorough: on outcome variables / regressors as well as on model specification and outliers, B: Some sensitivity analysis (eg. on regressors), C: No) See Additional file 2. *The category longitudinal/TSCS would include both studies that follow a sample of individuals over time, and those that include repeated observations at the country level, studying change over time with the country as unit of analysis

Given the complex nature of the topic and the intrinsic impossibility in carrying out intervention studies, we found that rating the quality of studies was not only extremely difficult but also potentially risked over-simplification. For this reason, we have provided a methods assessment using five criteria (see Additional file 2: Type of evidence). It should be noted, however, that in this context, different types of study can provide complementary evidence, and that this classification reflects different ‘types of evidence’, rather than overall quality.

We present the results following the structure of the framework (Fig. 1) concerning trade, investment, socioeconomic dimensions, such as global flows of information, and political aspects and their impacts on nutritional outcomes. We also comment on the differential results across population groups, defined by the main socioeconomic variables, which moderate the impacts of globalization.

### Economic globalization: Trade and investment

Six of the studies reviewed used index measures of economic globalization [55–58] [51, 59], which include flows of goods, services and investment as well as barriers to trade and investment. Three of these studies find that economic globalization tends to reduce obesity and overweight [51, 55, 56] as well as caloric and fat intakes [56] although the effects are small [55] or non-significant after controlling for additional variables such as urbanization, food prices, female participation in the workforce or number of McDonalds per capita [56], which can reflect potential confounding but might also be capturing partial impact mechanisms [55].

The remaining three studies find that economic globalization has a negative impact on nutrition-related health outcomes, leading to increased diabetes [57], overweight and obesity [59] and increased BMI [57, 58]. Oberlander et al. [57], find that, despite associations with diabetes prevalence and BMI, there seems to be no significant impact of economic globalization on dietary patterns.

The apparently contradictory findings can most likely be attributed to a certain extent to differences in the data. Oberlander et al. [57] use the longest time series, including data on 70 countries for 40 years, while de Soysa et al. [51] use the largest number of countries, including data on 180 countries for 23 years while Costa-i-Font et al. [55] include only higher income countries.

Moreover, studies differ in terms of the approach to estimation and methods chosen to deal with potential confounding effects. Schram et al. [59] use System Equations Modelling (SEM) to carry out pathway analysis on cross-sectional data, Costa-i-Font et al. [55] and de Soysa et al. [51] use panel corrected standard errors, which is a method to account for heteroskedasticity in time-series-cross-section data. Oberlander et al. [57], meanwhile, use group standard errors and a five-year lag on the main explanatory variables. Finally, while some key control variables such as income, inequality and urbanization are included in all studies, there are differences in terms of additional control variables, which can modify the interpretation of results (for example, Schram et al. [59] account for tobacco consumption, while de Vogli et al. [58] control for poverty rates).

Overall, the results regarding economic globalization as a whole are inconclusive. The inconsistencies both across and within studies suggest that the association between economic globalization indices and nutritional outcomes is complex and easily confounded or captured by simpler variables. Studies looking at aggregate indices are relevant, however, in highlighting the importance of aspects of globalization not captured by the economic component of the index, including flows of information or political, policy and regulatory space, which we discuss in Section “Policy and regulatory space”.

### Trade

We identified 11 studies analysing the nutritional impacts of trade openness or reduction of trade barriers. Controlling for a wide range of variables including GDP, income levels, urbanization and other socioeconomic variables such as occupation and household structure, these studies find mixed results concerning undernutrition, with some recent evidence suggesting that trade openness might be associated with reductions in underweight and increases in nutrient supply and intake and various proxies for dietary quality. There is no convincing evidence linking trade openness to increased overweight, obesity or other measures of diet-related NCDs.

Three early studies based on country-level data found a negative association between dependence on non-service or primary exports and average per capita availability of calories and especially proteins in the Latin-American context [60] and for developing countries in general [61, 62]. This negative relationship was attributed partly to the restrictions to imports including quotas and other non-tariff barriers that frequently accompanied export-promotion policies [60]. These studies, however, found the impacts to be small compared to the effects of foreign investment [61] or insignificant after controlling for investment and other economic variables [62]. Moreover, Jenkins and Scanlan [62] found that dependence on primary exports had no impact on child underweight.

Six studies analysed the relationship between overall trade openness and dietary patterns, underweight or BMI. Bezuneh and Yiheyis [63] found that the removal of trade barriers was associated with short-term falls in nutrient availability per capita, with positive longer-term effects and insignificant “net” impacts. However, this study, is based on a relatively small sample, compared to more recent studies [64].

Del Ninno, Dorosh, and Smith [65] used a quasi-experimental approach, comparing three episodes of severe floods in Bangladesh. They found that, in the absence of private imports, per capita calorie intake of the rural poor would, measured at the household level, have decreased significantly due to scarcity and increased prices of rice. The authors find, however, that public interventions in price regulation and transfers also played an important role in mitigating hunger following natural disaster episodes.

Based on more recent data, three studies have found that trade openness and tariff reduction are associated with increased calorie availability per capita [66], improved aggregate indicators of dietary diversity and quality [64], and decreased odds of being underweight for both rural and urban men and women [67]. The latter study, however, is based on cross-sectional household-level data, so further research would be needed in order to determine whether this association might be causal. Neuman et al. [68], meanwhile, found no evidence of a significant association between mean tariff rates and mean BMI or underweight in a multi-level multi-country analysis of 30 LMIC, although they found that higher tariff rates were associated with lower BMI for poorer, rural populations.

Overall, neither trade as a proportion of GDP or tariff levels seem to be directly associated with increased prevalence of overweight, obesity or NCDs. In the study by Nandi et al. [67] the association between trade openness measured through tariff levels and overweight, unlike the association with underweight, was found to be insignificant. Miljkovic [69] report positive impacts of trade on obesity rates in a fixed-effects model controlling for country heterogeneity but not income, urbanization or inequality. The same study reports non-significant effects of trade openness on adult obesity rates at a country level using a quantile regression model. Perhaps more surprisingly, de Soysa and de Soysa [51] report a negative association between trade openness and rates of overweight for children and adolescents. The authors argue that if globalization increases the returns to labour this could increase the incentives to invest in children’s health, leading to healthier diets and reduced levels of obesity and overweight.

### Foreign direct investment

Overall, studies analysing the role of FDI suggest that FDI might be associated with an increased consumption of sugary and highly processed foods and increases in overweight and obesity in LMICs in particular. Four studies found positive associations with obesity, overweight or related dietary indicators, one found a positive association which was nevertheless not robust to changes in model specification [69], and three studies found non-significant associations.

Schram [70], using a natural experiment design, found a significant increase in sugar-sweetened beverages sales per capita, attributable to the removal of restrictions to FDI in Vietnam. Baker et al. [28] used a similar approach in Peru and found that following trade and investment liberalization that significantly increased FDI inflows, sales of carbonated drinks stagnated, while sales of juice, energy and sports drinks, as well as bottled water, increased. These more nuanced results emphasise the role of branding, diversification of branding and preference change, which can lead to changes in demand towards juice and sports drinks, which are often high in sugar and energy content, but marketed as healthy, potentially reaching a wider consumer base [71]. These findings corroborate previous research by Stuckler et al. [72] who showed that levels of FDI moderate the impact of GDP on consumption of unhealthy food products, including soft drinks, ice-cream, and confectionery, ultra-processed and packaged foods.

Miljkovic et al. [69] used a quantile regression specification with country-level panel data, finding that FDI was associated to increase obesity rates only in LMICs, although the association was insignificant in their fixed effects specification including all countries. In a multi-level analysis of adults in LMICs, Nandi et al. [67] found that FDI was associated to increased prevalence of overweight for rural men only. The same study found no association with prevalence of underweight.

However, Neuman et al. [68] and de Soysa and de Soysa [51] find no significant associations of FDI with overweight and obesity, while Sudharsanan et al. [73] find that the impact of FDI on the prevalence of diabetes is insignificant after controlling for population ageing.

The discrepancies regarding the significance of effects might be due to the differences in the data coverage (Miljkovic et al. [69] use a smaller number of countries than de Soysa and de Soysa [51] or Sudharsanan et al. [73], for example, but a longer time period) and study design (Miljkovic et al. [69], for example only find significant associations when using a quantile regression design, which is not implemented in other studies).

Although there appears to be some evidence of an association between FDI and some indicators of dietary quality, we have found no evidence linking it to underweight or undernutrition. The earlier literature analysed this issue within the debate on the “dependency versus modernization” impacts of foreign investment and Trans-national Company (TNC) penetration in developing countries. Two studies [61, 74] found strong negative impacts of TNC investment on per capita availability of calories and proteins in LMICs, while Jenkins and Scanlan [62] find a positive association which is small compared to the effects of domestic investment. More recent studies [75, 76] added some nuance to this debate, showing that the impact of FDI on nutritional indicators seems to vary depending on the sector. The former study concluded that FDI in the primary sector has tended to harm food security in LMICs through a combination of resource exploitation, labour market effects and negative environmental and demographic externalities. However, FDI in the manufacturing sector leads to modernization, technological and human capital spill-overs and increased wages, improving nutritional outcomes. The negative impact of agricultural FDI on calorie and protein intakes is corroborated by Djokoto [76] in the case of Ghana. Three studies were identified that examined explicitly the relationship between FDI and underweight, all of which failed to find any significant association for either adults [67, 68] or children [62].

### Sociocultural aspects of globalization

Five studies analysed the impact of social components of globalization alongside economic components [51, 55–57, 69]. Social components include flows of information via television (TV), internet and telephone, interpersonal contact and cultural aspects. The first two of these studies [55, 56] find that globalization as a whole tends to be associated with an increase in obesity rates, and this effect is driven largely by the social component. This is consistent with findings by Miljkovic et al. [69] who find that social globalization leads to higher prevalence of obesity. Oberlander et al. [57] find that, while economic globalization is associated with a higher prevalence of diabetes and higher BMI, only social globalization is associated with increased supply of sugar and animal protein, with the results being primarily driven by increased flows of information (e.g. through internet and TV). de Soysa et al. find non-significant impacts of social globalization on the prevalence of obesity [51], in a model that controls for the economic globalization component of KOF index and the standard control variables, as well as including country and time fixed-effects.

Further research is needed in order to interpret these findings in the context of food systems and nutrition outcomes, examining the impacts of specific variables within these indices. Although these studies did not report strong multi-collinearity across the control variables, the complexity of the mechanisms involved and the potential inter-relations between the variables and indices included should be taken into account when interpreting these results.

### Policy and regulatory space

Three studies analyse the nutritional impacts of political and policy changes underlying globalization processes, comparing these to the effects of economic integration processes using the political component of KOF index, as well as an Index of Economic Freedom [51]. Goryakin et al. [55] suggest that there is a positive and convex relationship between political globalization, measured by the KOF index, and overweight. This implies that the association is not proportional and does not tend to plateau as integration increases, but tends to be larger at higher levels of political integration. De Soysa et al. [51], on the other hand, using a larger sample, find that both political globalization measured through KOF index, and the degree of free-market capitalism, measured through the Economic Freedom Index, seem to be associated with reduced rates of child and youth obesity. Costa-i-Font et al. [56] check for the effects of political globalization as part of their sensitivity analysis, finding no significant impacts on obesity or calorie intake, although there seems to be an association with higher fat intakes.

The quantitative studies in this review offer limited evidence on the direct impact of policy and regulatory changes associated with trade and investment liberalization, suggesting some potential associations that deserve further analysis, but overall leading to mixed and inconclusive findings. The differences in results, as in other cases, can be attributed both to data coverage as well as potentially to the study design and choice of control variables. de Soysa et al. [51] use the largest country sample, while Goryakin et al. [55] include additional controls such as the Human Development Index (HDI) in all of their fixed-effects specifications, where country heterogeneity is controlled for.

### Socioeconomic and demographic factors as moderators of impact

Only four articles were found to control for individual level factors [55, 65, 67, 68]. Of these, only three estimate differential associations of globalization or macroeconomic variables with nutrition outcomes in different subgroups. Two of these studies found significant differential effects across sub-groups. Nandi et al. [67], for example, find that increased FDI is associated with a 17% increase in the odds of overweight for rural men only. Neuman et al. [68] find that, although FDI is positively associated with overweight in most sub-groups, the association is negative for the wealthiest urban category, which is consistent with market segmentation practices whereby healthier products are targeted at high income consumers. de Soysa and de Soysa [51] is the only study focussing on children and youth. The authors comment that impacts on adults, included as part of their sensitivity analysis but not reported, are very similar to those obtained for individuals under the age of 19.

## Discussion and interpretation

The empirical evidence analysed in this review highlights the important role of globalization processes as drivers of dietary change and nutrition-related health outcomes. There is no agreement, however, with respect to the overall impacts of economic globalization and its components, or even the sign of these impacts, as discussed in Section Economic globalization: trade and investment. Results can be affected by the type of countries included (LMIC only [67], versus panels including both high and low income countries [69]), the population studied (children and youth [51], women only [55], adults only [56], or the overall population [73]), the choice of control variables (for example, whether the study controls for inequality, HDI or female labour participation), as well as the method chosen to control for heterogeneity (both time invariant and dynamic, [57]) and to capture non-linearities [55] and interactions across factors [72].

The studies reviewed have some limitations which should be considered when interpreting our results. Seven of the articles identified rely on average nutrient per capita availability at a country level, which has been found to be a weak indicator of important nutritional outcomes such as child underweight [62]. More generally, the use of aggregate indicators of nutrition can mask the uneven distribution of the gains of liberalization, or hide important sectoral differences, which deserve further investigation. The use of quantitative, a posteriori statistical analysis, moreover, precludes the analysis of some country-specific mechanisms and their interactions. Furthermore, we should be cautious when drawing conclusions on causality, given that these studies are based on observational data (often highly aggregated), and some of the methods used might be better suited for the analysis of broad trends and associations. Although these limitations can be addressed to a certain extent through careful study design, the results from the studies in this review should be interpreted with caution and should be understood as complementary to other types of evidence, both quantitative and qualitative.

Evidence on the associations between globalization processes on undernutrition and underweight is limited, particularly compared to the number of studies analysing overweight and obesity. There is a scarcity, of empirical studies, based on cross-country or natural experiment designs which control for confounding factors and which use individual or household level measures of dietary adequacy and nutritional status including nutrient deficiencies, underweight and stunting.

Despite these limitations, the studies reviewed, particularly when analysed together, provide relevant insights regarding different mechanisms and sub-components, their relative importance, distinctive roles and potential interactions. First, the suggestion that trade openness and FDI is likely to have played distinct roles so far in the nutrition transition. There is some recent evidence linking traded openness to reductions in underweight, [65, 67] and improvements in dietary adequacy and diversity [64] but not to increased prevalence of overweight or obesity [51, 67, 69]. FDI, meanwhile, has been found to be associated with increased prevalence of obesity and overweight in LMICs [28, 67, 69, 70], (although not diabetes, according to the study by Sudharsanan et al. [73]) but there is no clear evidence that it is associated with reductions in undernutrition. Mihalache et al. [75] and Djokoto [76] find that the impacts can depend on sectoral composition and context-specific mechanisms relating to migratory and labour market dynamics.

This pattern of association could reflect a trend towards FDI as the main vehicle for food system integration, which has been identified and described in the literature [28, 77]. FDI can provide greater opportunities for market penetration of TFC through vertical and horizontal integration, transformation of the distribution and retail segments, effective advertisement and adaptation to local consumer tastes or ‘glocalization’ [78].

The lack of association between trade openness and over-nutrition could also suggest that availability and affordability of food products, per se, are not enough to lead to the changes in lifestyle and consumption patterns associated to NCDs prevalence. Direct investment, on the other hand, has the capacity to deeply transform the food sector and the wider economic system, altering consumer behaviour as part of this process (see Section Foreign direct investment).

Additionally, the (relatively scarce) evidence linking trade openness to reduced under-weight or improved dietary quality should be interpreted with caution. It is important to bear in mind that in this review we do not include outcome measures related to food prices or relative food expenditure which might be affected by trade liberalization. Short-term relative price fluctuations, however, can have important impacts on food security which might not be captured by the studies reviewed.

The apparent association between trade openness and improved nutrition outcomes, however, could reflect the impact of trade policies explicitly aimed at improving food security and mitigating the impact of international price spikes on domestic prices of staple foods. These measures include selective reductions in import protection of essential foods, sometimes coupled to public stockpiling and distribution programs [79]. Despite the controversy around the effectiveness of some of these interventions and their impacts on global price volatility [80], measures aimed at selectively lowering import barriers for food staples have been found to be successful in several LMICs [25, 79, 80].

Policy makers can also exert control over FDI and transnational food companies, setting standards for processing, labelling, packaging and retail. Once large investors enter the market, however, food systems are rapidly and deeply transformed in ways that can be hard to control, requiring regulation at many segments along the value chain, from processing to packaging, advertising and distribution [81]. Moreover, some have argued that, as large companies become established nationally, they can constrain the space for nutrition oriented policy through lobbying and re-location threats [82].

The lack of apparent overall association between FDI and under-nutrition can be interpreted as evidence that the most disadvantaged segments of society are excluded from the potential benefits of economic growth in general, and of more efficient and modernized food systems in particular. In addition to their low purchasing power, these populations often live either in poor quality housing or slums which have little infrastructure [83], or in remote rural areas, providing few economic incentives for the establishment of supermarkets and the delivery of a variety of fresh produce.

The multi-country studies in this review generally measure aggregate flows of FDI at a national level. In terms of its association with overweight and obesity, after controlling for a range of socio-economic variables, this aggregate FDI is generally interpreted as a proxy for greater integration of food systems, and the entry of TFCs into the market [72]. While this might be a reasonable assumption in most cases, FDI has deep impacts on the productive and social structure of receiving countries that go well beyond food systems, affecting income distribution, migration patterns and lifestyles, all of which can have important implications for nutrition outcomes [75]. The detailed sectoral analysis of the impacts of FDI on nutrition deserves more attention. A combination of case studies and cross-country analysis might shed more light over complex context-specific mechanisms concerning FDI in the primary, secondary and tertiary sectors.

Another relevant finding in the literature concerns the potentially crucial role of sociocultural aspects and in particular global flows of information in explaining dietary changes. The empirical literature uses the social component of the KOF index of globalization which, among others, includes variables reflecting TV ownership, internet access, foreign films viewing, use of phones and number of McDonalds per capita. Two studies find relevant positive associations with overweight, calorie and fat consumption, which seem to dominate the effects of economic flows [55, 56]. These results offer more than one interpretation, however. On the one hand, the access to communication technologies and foreign entertainment products can lead to increased exposure to globalized food marketing, which has been identified as a key component of food system integration. Marketing includes not only conventional advertising but also sports sponsorship and product placement in films, videos and other forms of entertainment [36, 84]. Moreover, advertising can have indirect effects on diets, as it increases the demand not only for the marketed brand but for the category as a whole, be it snacks, bakery products, fries or hamburgers. The variable reflecting number of McDonalds per capita is part of the “cultural proximity” sub-component of the index. In this context, this variable could potentially be interpreted as a food-specific proxy for FDI influx, and one which epitomises the subordination of the exchange of information and cultural values to economic forces. On the other hand, increased access to technology could be correlated to other changes in lifestyle, social-relational characteristics of labour and socialization, which could lead to changes in dietary patterns, as discussed in Section Interaction with socioeconomic drivers of nutrition. This is a relatively under-studied mechanism, however, and further research will be necessary in order to disentangle the potentially overlapping mechanisms connecting increased interconnectivity and information flows to changes in nutrition outcomes.

Finally, the evidence suggests that globalization processes have different impacts across sub-groups, without necessarily exhibiting a continuous gradient. This is consistent with the dynamics of market segmentation, which tends to create divergent dietary patterns within countries, with healthier products being targeted towards wealthy urban consumers, while lower income groups become the target consumers for calorie dense “junk foods” [6].

The existence of important differences in impact across groups can also be a product of interactions between mechanisms, which either compensate or enhance each other’s effects. For example, FDI might increase the access to unhealthy food commodities, but associated income growth and increased access to information might compensate by promoting health-seeking behaviour. Conversely, longer working hours or reduced time available for cooking might exacerbate the impacts of changes in food environments. Further analysis of group-specific impacts of trade and investment policies can be useful when it comes to developing more effective policy interventions.

## Conclusion and implications for policy and research

Our results indicate that, overall, globalization processes and the trade and investment policies underpinning them have so far played an important role in driving changes in the nutrition status of populations in high, middle and low-income countries. Empirical literature provides, however, a nuanced view of the impact of globalization on nutrition, indicating that different processes and sub-components have different effects. In particular, trade openness contributes to shifts in dietary patterns, increasing dietary diversity and availability of cheap calories and fats and, on average, reducing under-nutrition. However, trade openness is not sufficient, per se, to explain the increases in obesity and overweight. These seem to be more associated to FDI and global flows of information in LMIC, including food marketing and advertisement.

Moreover, sociocultural aspects and particularly information flows seem to have an important impact on dietary patterns, overweight, obesity and consumption of calories and fats, even dominating the effect of trade and investment flows. This could reflect the impacts of exposure to globalized marketing, or it could reflect other lifestyle changes associated with the use of new communications technologies.

The studies reviewed support the view, suggested by others [12, 56] that neither overall protectionism nor unregulated liberalization are likely to reduce malnutrition, making adequate monitoring and intervention a necessity to avoid negative impacts of globalization processes on nutrition. In addition, our results suggest that governments do not necessarily face a trade-off in dealing with the double-burden of malnutrition (liberalize, and reduce under-nutrition, but face increases in over-nutrition and chronic disease, or protect against the latter, at the risk of increasing food insecurity). Rather, governments can in principle play an important role in prioritising food security through nutrition-sensitive trade policy, while simultaneously controlling and regulating foreign investment and marketing in the food sector, in order to avoid the creation of obesogenic environments. In this sense, the potentially constraining impacts of trade agreements on the policy space to pursue public health objectives have been identified as an important pathway for trade liberalization impacts on nutrition, which remains relatively unexplored in the quantitative literature [12]. Furthermore, the existence of significant differences in impacts across population sub-groups, where the most vulnerable populations tend to be affected disproportionately, highlight the need to reduce inequalities in access to food, and to develop targeted policies which can address the needs of those groups which might be most vulnerable to the impacts of globalization.

Given the complexity of the topic and the high susceptibility to bias, thorough and transparent sensitivity analysis regarding outcome measures, control variables and study design is important in order to advance the debate and improve comparability across studies. Although different approaches can provide complementary evidence, more studies are needed that use natural experiments or other methods to control for confounding and reduce bias. The roles of sociocultural, lifestyle and political aspects of globalization in the nutrition transition are relatively understudied in the quantitative literature and might be fruitful areas of research. Analyses based on overall indices of globalization can provide relevant insights but are often hard to interpret [85]. As suggested in recent studies [51], more evidence is needed on the impact of specific sub-components of wider processes of liberalization, including sector-specific FDI flows or different types of trade barrier. Further research on this topic should also attempt to incorporate measures of stunting, wasting and micronutrient malnutrition. Perhaps more importantly, research is needed to improve the current understanding of differential impacts of globalization and liberalization processes across sub-groups of population, in order to identify potentially vulnerable groups.

## Additional files

Additional file 1: Search strategy. Contains the search strategy for the review. (DOCX 17 kb)

Additional file 2: Type of evidence. Contains assessment criteria. (DOCX 12 kb)

## Footnotes

1 See Dreher et al. (2006) [43] for a detailed description of KOF index of globalization. The economic component includes flows of international goods, services, investment and capital, as well as restrictions, such as tariffs or other taxes on international trade as well as hidden import barriers (in the form or regulations and standards, for example). The political component includes number of embassies, membership in international organizations and participation in “UN security council” meetings. The social component includes measures of flows of information (through internet, television, newspaper and other channels), cultural proximity and personal contact (including measures such as number of foreign residents, tourism or costs of a call to the US). The different components and sub-components of the index and their potential interpretation in the context of our study will be further discussed in the Results section.

2
https://www.wto.org/

3 For more detailed information see the WTO register of regional and bilateral trade agreements as notified to the organization https://www.wto.org/english/tratop_e/region_e/regfac_e.htm.
