# Supplementary material for: Economic globalization, nutrition and health: a review of quantitative evidence
Source: Global Health. 2019 Feb 20;15:15. doi: 10.1186/s12992-019-0456-z (PMC6381642; doi:10.1186/s12992-019-0456-z)
Supplement: Supplementary file 1 — Search strategy. Contains the search strategy for the review. (DOCX 17 kb) [file 12992_2019_456_MOESM1_ESM.docx]

### Search Strategy

1. Economic globalisation; ti ab

TS=("trade polic*" OR "trade agreement*" OR "trade liberali*" OR "World Trade Organization agreement*" OR "free trade" OR "investment treaty" OR "trade treaty" OR FDI OR Foreign Direct Investment” OR trade openness OR "economic globali*" OR "KOF Index" OR "Maastricht Index" OR "G-Index" OR WMRC OR ATK OR KFP)

1. Food environments; ti ab

food* OR bread OR cereal* OR condiment* OR candy OR chocolate OR dairy OR eggs OR fruit OR honey OR meat OR cheese OR rice OR maize OR flour OR wheat OR corn OR sugar OR coffee OR cocoa OR nut* OR seed* OR vegetable* OR legume* OR bean* OR beverage* OR drink* OR soda* OR juice* OR fat OR oil OR sweet* OR fish OR seafood OR milk OR cream OR "soy* beans" OR "energy drink*" OR "soft drink*" OR "grocer*"OR supermarkets OR "convenience store*" OR snack* OR "farmer* market*" OR "cafeteria*" OR "vending machine*" OR restaurant* OR meal* OR "corner store*" OR "corner shop*" OR "wet market"

1. Nutrition/health; ti ab

TS=(diet* OR nutrition* OR malnutrition OR nutrient* OR macronutrient* OR micronutrient* OR kilojoule* OR "energy intake" OR calorie* OR protein OR carbohydrate OR fibre OR fiber OR sugar OR vitamin* OR mineral* OR underweight OR overweight OR obes* OR "body mass index" OR BMI OR height OR weight OR stunting OR "growth retardation" OR "chronic disease" OR "non-communicable disease" OR NCD OR diabetes OR "cardiovascular disease" OR "heart disease" OR stroke* OR "kidney disease" OR "renal disease" OR "cancer" OR hypertension OR "blood pressure" OR hyperglycaemi* OR "blood sugar" OR "blood glucose" OR cholesterol OR hypercholesterolaemia OR morbidity OR mortality OR "disability adjusted life years" OR DALYs OR health OR malnutrition OR undernutrition OR malnourished OR wasting OR death*)

1. Quantitative, retrospective studies.

Quantitative OR quantif* OR "estimat*" OR "statistic*" OR "econometric*" OR "correlat*" or "control* for" OR "empiric*" OR "cross-section*" OR "cross section" OR "time-series" OR "time series" OR "panel" OR "natural experiment*" OR "difference*in*difference" OR regress*

1. 1 and 2 and 3 and 4
