# Supplementary material for: Economic globalization, nutrition and health: a review of quantitative evidence
Source: Global Health. 2019 Feb 20;15:15. doi: 10.1186/s12992-019-0456-z (PMC6381642; doi:10.1186/s12992-019-0456-z)
Supplement: Supplementary file 2 — Type of evidence. Contains assessment criteria. (DOCX 12 kb) [file 12992_2019_456_MOESM2_ESM.docx]

| **Type of evidence** | **Design** | **Statistical analysis** | **Type of outcome variable** | **Data** | **Sensitivity analysis** |
| --- | --- | --- | --- | --- | --- |
| A | natural experiment | Structural equation modelling. Reduced-form regression, time-series analysis, other | Uses several related outcome variables including prevalence or status of dietary-related disease as well as relevant proxies | Both individual and country-level outcome variables | Thorough: on outcome variables / regressors as well as on model specification and outliers |
| B | Longitudinal/  time-series-cross-sectional (TSCS)/  Time-series | Simple one-on-one correlation | Nutrition outcomes: Prevalence/status of diet-related disease (CVD, diabetes or others). Relevant biomarkers (obesity, underweight or overweight, BMI) | Individual-level outcomes | Some sensitivity analysis (eg. on regressors) |
| C | Cross-sectional | Descriptive | Context-relevant proxies for nutrition outcomes: per capita consumption of key foods/nutrients | Country-level outcome variables | No |

*The category longitudinal/TSCS would include both studies that follow a sample of individuals over time, and those that include repeated observations at the country level, studying change over time with the country as unit of analysis.
